# The Public Health Service: Its Wide Field, Needs and Future

**Published:** 1916-09-02

**Authors:** 


### THE PUBLIC HEALTH SERVICE.

### Its Wide Field, Needs and Future.

It has been said that all our plans should be made with a view to the state of war becoming the basis of our whole national, social, economical, and industrial life. The Public Health Services of the country, depleted though their ranks have been by the exigencies of medical war work, have modified rather than suspended their activities. In providing for present conditions the needs of that much discussed future "after the war" are being steadily kept in view.

Recent public health developments and legislation have shown a remarkable trend towards the supervision and protection of the mother, the infant, and the child. The notification of measles and of ophthalmia neonatorum, the amendment of the adoptive Notification of Births Act of 1907 by the Act of 1915, the schemes for maternity care and child-welfare, and even, in some degree, the Venereal Diseases Regulations, all aim at securing a healthy infancy and childhood for the race. It is no longer a surprise to find the Board of Education taking a place, and co-operating with the Local Government Board, as a health authority. The public health work of the State is not concerned only with groups of people and with epidemiology. Preventive medicine and curative medicine are, as has been exemplified in the working of the tuberculosis sections of the National Insurance Act, in practice inseparable. Public health work will, in the future, whatever may be the ultimate range of the State Medical Service, deal more and more with the individual, and especially with the child, both in health and in disease.

The further expansion of the Public Health Services is inevitable and cannot but be rapid. At first sight it would seem that the financial burdens of the nation will be for many years to come of such magnitude that expenditure will need to be curtailed and progress to be stayed. That public health developments will be arrested we do not believe. Local authorities will, no doubt, hesitate to take up large loans and launch extravagant building schemes, but the interests of the coming generations will be paramount, and perseverance in carrying out altruistic public health measures will prove to be not inconsistent with true economy. Much of the activity, expenditure, and material which have been necessitated by, and called into being as a result of, the war will later be diverted into other channels and will be available to further fresh interests: buildings, medical and nursing skill, experience in after-care may be adapted to minister to the needs of the public health as to those of wounded soldiers.

Among the medical *personnel* of the Public Health Services, county medical officers, medical officers of health, tuberculosis and school medical officers, etc., must be included the doctors of the Poor-Law Medical Service. The Poor-Law Medical Service has remained distinct, the conclusions and recommendations of the Poor Law Commission notwithstanding. The unique opportunity of the passing into law of the National Insurance Act, the grant of old-age pensions, the enlargements of the scope and authority of the County Councils and their medical officers, the increasing importance in the national economy of the great public infirmaries and asylums—all have come without that necessary union into one co-ordinated scheme of public assistance which should, and which ultimately must, do away with the anomalies, the overlapping, and the waste of our multiple petty "authorities." The aims of the Public Health Services are sure and sound, and the brilliant future of State medicine is assured, but a further and closer association between public health work, education, and medical treatment has long been needed and is becoming increasingly necessary. Foremost amongst changes in administration must come Poor-Law reform.

The Public Health and Poor-Law Medical Services, as we must at present distinguish them, offer, and will continue to offer in increasing number, attractive opportunities of employment to medical men and women. Appointments in the Services give no prospect of pension in most cases, and are (with the exception of some of the higher administrative posts) not highly paid, especially considering the expenditure of time and money which applicants have incurred in obtaining diplomas in public health or degrees in State medicine. Only too often, moreover, there is no security of tenure of office. Such appointments, however, afford interest and valuable experience, and will, it may confidently be hoped, improve both in prospects and in commanding salaries which will secure public medical officers of the highest standard of education and ability.

